# Promoting Water Conservation Based on the Matching Effect of Regulatory Focus and Emotion

**DOI:** 10.3390/ijerph18041680

**Published:** 2021-02-09

**Authors:** Xiaomei Wang, Lin Zhang, Xiaoyu Jiang, Jia Wang

**Affiliations:** School of Media Studies and Humanities, Zhejiang University City College, Hangzhou 310015, China; wangxiaom@zucc.edu.cn (X.W.); liangshu0323@163.com (L.Z.); jxyzucc@Outlook.com (X.J.)

**Keywords:** regulatory focus, emotion, water-saving information, fit

## Abstract

This study aimed to examine the effects of regulatory focus and emotions on water-saving information dissemination. The findings revealed that when water-saving information is framed with a prevention focus, sad emotion fosters more active willingness to engage with the information dissemination than cheerful emotion. However, a promotion focus coupled with cheerfulness is slightly more persuasive than a promotion focus coupled with sadness. Furthermore, compared to the individuals in the nonfit group of emotions who had a regulatory focus, the individuals in the fit group formed a more favorable water-saving attitude and demonstrated a slightly higher willingness to disseminate water-saving information. This article is the first to contribute to exploring the dissemination of water-saving information from the perspective of the interactive effect of individual cognitive motivation and emotion.

## 1. Introduction

Water is an indispensable element for human survival, as well as a basic industry and strategic resource for national and regional development. With changes being made in regard to time, space, and climate, water problems are becoming increasingly complex and changeable. In particular, problems such as shortages of water resources, frequent water disasters, serious water pollution, deterioration of the water environment, and drought and rainstorms pose great threats to human survival and development. Inadequate water resources cause the significant health and socio-economic consequences of poor nutritional status [1]. Since the beginning of the 21st century, the process of globalization has accelerated rapidly. As the global population continues to grow, freshwater resources continue to decrease, and the serious waste of water resources and other water crises has aroused people’s concern, and the water crisis has become a major issue concerning the life and death of human beings. In facing these challenges, experts and commentators have called for a range of water-saving measures, such as technical improvements, discounts on water-saving products, an end to the dumping of all waste into the sea, the recycling of water, and regulations to increase domestic water savings [2,3,4]. In Cape Town (South Africa), the infamous drought of 2015–2017 offers important lessons for water management [5]. Environmental protection is an effective way for humankind to prevent water crises and save its own destiny. Despite the importance of water conservation, little direct research exists on how emotional and motivational factors might influence the persuasiveness of water conservation intervention messaging.

One public service announcement persuades people with the claim that “wasting water will turn the earth into a desert and creatures will be extinct.” Another claims that “saving water makes the earth a green home and brings benefit to humans.” In one way, these two announcements both persuade individuals to conserve water. However, in another way, they are very different from each other. The former announcement focuses on the hassles of wasting water, and it emphasizes the costs of not adhering to conservation behaviors, while the latter highlights the advantages of saving water, and it stresses the benefits of sticking to conservation behaviors. Thus, these announcements represent two different information frameworks. Some information frameworks emphasize positive results, and some emphasize negative results. Which frame of expression is more persuasive? There is still no clear or consistent answer to this question found in previous relevant studies. Some researchers believe that individual differences are important in influencing variables that affect the information framing effect [6,7]. Cesario et al. (2013) incorporated framing and individual differences into a single framework. This framework describes multiple levels of self-regulation of information representations. The authors claimed that the information framework can initiate regulatory orientation and that different information content can lead to different regulatory orientations. Specifically, information related to obtaining, for example, winning, can induce a promotion orientation, whereas information related to loss, such as losing, can activate prevention orientation [8,9,10]. Moreover, researchers have found that gain-framed messages that emphasize achievement concerns (i.e., inducing a promotion focus) are more effective than those that emphasize safety concerns (i.e., inducing a prevention focus). However, the reverse has also been found to be true; i.e., loss-framed messages that emphasize safety concerns are more effective than ones that emphasize achievement concerns [6].

There is a symmetry present in that the individuals with a promotion focus are more primed by pleasure and the individuals with a prevention focus are more primed by pain [11]. Cesario et al. (2013) found that information represented by persistence in regard to a certain behavior to obtain cheerfulness is much more persuasive for promotion-focused people, while information represented by a lack of persistence in regard to a certain behavior to experience pain is much more persuasive for prevention-focused people. In other words, the effect of information persuasion depends on whether the outcome of the behavior is security-related (prevention orientation) or growth-related (promotion orientation); in addition, emotions play a role in this process. Specifically, a gain-framed message might be more persuasive for people with a promotion focus who are in a state of cheerfulness, while a loss-framed message might be more persuasive for people with a prevention focus who are in a state of sadness. In this research, we examine how the effects of emotions on water conservation behavior vary as a function of different regulatory concerns in gain- or loss-framed messages.

This paper consists of five sections. Section 1 introduces the significance and background of the research. Section 2 reviews the literature on regulatory focus, emotion, and altruistic behavior and develops research hypotheses. Section 3 presents the research methodology and the results of study 1, which investigated the effect of price discounts on perceived savings based on regulatory focus and quality uncertainty. Section 4 presents the research methodology and the results of study 2, which investigated the effect of value-added promotions on perceived savings based on regulatory focus and quality uncertainty. Finally, Section 5 draws the conclusions of this paper, including theoretical and practical implications and limitations.

## 2. Literature Review and Hypotheses

### 2.1. Regulatory Focus

Higgins (1997) proposed regulation focus theory and believed that people have a tendency to move towards a certain type of goal [9]. The outcome of an objective can be conceptualized into four types, namely, gain, non-gain, loss, and non-loss. The gains and non-gains in connection with the gain target can induce a promotion focus, while the losses and non-losses in connection with the loss target can induce a prevention focus. Promotion-focused individuals pursue pleasure and tend to be more sensitive to the presence or absence of positive results. In contrast, prevention-focused individuals avoid pain and tend to be more sensitive to the presence or absence of negative results [12,13].

Individuals with different regulatory foci show significant differences in their thinking, cognition, and information processing [14]. Promotion-focused people tend to adopt a strategy of approach, they are eager to maximize their profits, and they believe that things are developing in a benign way and do not need special actions [15]; therefore, they tend to adopt heuristic strategies in regard to their decision-making [16]. In contrast, prevention-focused people mainly adopt a strategy of avoidance, they are focused on avoiding losses [17], and they believe that problems will occur; therefore, action is needed to reverse the situation [15]. As a result, they tend to adopt systematic strategies and carefully avoid unexpected results [18], and they seek a great deal of information in regard to their decision-making [16].

### 2.2. Regulatory Focus and Emotion

Previous studies have found a close relationship between motivation and emotional experience [19]. Different emotions have different functions of regulatory foci. Cheerfulness promotes the function of eagerness [20]. When people are happy, they tend to pay attention to their current hedonic experience; pay more attention to aesthetic, experiential and hedonistic information; and avoid painful information [21]. In contrast, sad emotions promote the function of vigilance [20]. When people are sad, they are more vigilant and more sensitive to future losses, and they tend to pay attention to information that is instrumental and practical [22].

Scholars have also found that regulatory orientations are sensitive to specific emotions in different ways. Promotion-focused individuals more easily experience cheerfulness-related emotions, such as happiness (in the presence of positive outcomes), and dejection-related emotions, such as disappointment (in the absence of positive outcomes). In contrast, prevention-focused individuals more easily experience quiescence-related emotions, such as calmness (when negative outcomes are absent), and sadness (when negative outcomes are present) [9,23]. Regulating orientation and corresponding emotions can easily form a matching effect, resulting in a more positive attitude, which affects individual psychology and behavior [24]. The degree of dependence of regulating orientation on emotion is different; promoting orientation may encourage dependence on emotions, thereby making people tend to adopt perceptual and heuristic processing methods, while preventing orientation may hinder dependence on emotions. People tend to adopt both rational and systematic processing methods [15,16,17]. Thus, we predicted the following:

**Hypothesis** **1** **(H1).**  *Emotion plays a regulating role in the regulatory focus on, and the willingness or behavior towards, water-saving information dissemination.*

### 2.3. Regulatory Fit, Information Framework, and Persuasion

Regulatory fit theory (RFT) was developed on the basis of regulatory focus theory. This theory explains the relationship between an individual’s regulatory orientation and behavioral pattern in pursuit of his or her goals. When individuals with different regulatory orientations use their preferred behavioral patterns in pursuit of their goals, regulatory fit can be achieved. Promotion-focused individuals prefer to use eager strategies, while prevention-focused individuals prefer to use vigilant strategies [9,13]. Therefore, there are two fit conditions (promotion/eager or prevention/vigilant) and two nonfit conditions (promotion/vigilant or prevention/eager). Such a regulatory fit (or nonfit) produces feelings of rightness (or wrongness), which influences the way people process persuasive information [25]. Idson, Liberman, and Higgins (2000) found that people feel more motivated when the strategies they use match their regulatory focus [26]. Individuals are more likely to be persuaded when the information frame fits their regulatory orientation [27].

Regulatory focus theory plays an important role in understanding the framing effect of persuasion [28]. The information framework is one of the methods that is commonly used by researchers to prime state-based regulatory orientation. We use this framework to emphasize whether there are gains that activate the promotion orientation and to emphasize whether there are losses that activate the prevention orientation. However, while information can describe the results related to either promotion or prevention, sometimes, the semantically explicit terms “gains” and “losses” are not used [29]. For example, information describing a specific behavior (such as “eating fruit”) can produce gain-related results (such as “providing nutrition”), which are most effective for recipients who are promotion focused. However, the same information can also produce non-loss-related results (such as “preventing clogged arteries”), which are most effective for recipients who are prevention focused [6].

When the information contains elements related to the individual’s regulatory focus target, the individual often experiences a “natural fit,” that is, a regulatory fit. This fit can produce values. When the content of persuasion information contains elements related to the goal of promoting focus, that is, emphasizing the realization of positive results or the need for achievement, then the persuasion effect is better for individuals who are promotion focused. In contrast, when persuasion information emphasizes avoiding negative results or emphasizes safety needs and other factors related to prevention focus, the persuasion effect is better for individuals who are prevention focused. Researchers have conducted much related empirical research. For example, Idson, Liberman, and Higgins (2000) found that when people’s strategies match their regulatory orientation, they feel more motivated [11]. It has also been found that when the information framework matches participants’ regulatory orientation, then the participants are more likely to be persuaded [27]. For example, Spiegel, Grant-Pillow, and Higgins (2004) had participants read different framed messages (either a promotion-framed or a prevention-framed message), which encouraged them to eat more fruits and vegetables. The fit participants subsequently ate approximately 20% more fruits and vegetables over the following week than the non-fit participants [30].

Previous studies have found that a promotion focus is associated with feelings of cheerfulness (disappointment and anger) when promotion goals are (not) fulfilled. Relatedly, a prevention focus is associated with relief and relaxed feelings (fear, worry) when prevention goals are (not) fulfilled [31]. The process of goal pursuit is closely related to emotions, and there is a fit between these two factors. Moreover, the fit may produce a migration effect, which may subsequently affect the following tasks. Compared to a regulatory nonfit, a regulatory fit can strengthen an individual’s motivation, improve an individual’s task performance [30], improve an individual’s persuasion effect [32], and lead to a more positive attitude [24]. Therefore, the following hypotheses are proposed:

**Hypothesis** **2a** **(H2a).**  *Individuals who experience a regulatory fit (a promotion focus/cheerfulness, prevention focus/sadness) will show more favorable water-saving attitudes than will those who experience a regulatory non-fit (a promotion focus/sadness, prevention focus/cheerfulness).*

**Hypothesis** **2b** **(H2b).**  *Individuals who experience a regulatory fit (a promotion focus/cheerfulness, prevention focus/sadness) will show more favorable willingness to spread water-saving information than will those who experience a regulatory non-fit (promotion focus/sadness, prevention focus/cheerfulness).*

**Hypothesis** **2c** **(H2c).**  *Individuals who experience a regulatory fit (promotion focus/cheerfulness, prevention focus/sadness) will show more water-saving information dissemination behavior than will those who experience a regulatory nonfit (promotion focus/sadness, prevention focus/cheerfulness).*

## 3. Study 1

Study 1 was developed to directly examine the interaction of regulatory focus and emotion. We predicted that emotion would play a regulating role in the regulatory focus on, and the willingness or behavior towards, water-saving information dissemination (Hypothesis 1).

### 3.1. Method

#### 3.1.1. Participants and Design

Eighty-two participants were recruited from the Jiulian community in Hangzhou. Approximately 6.4% of the participants were 18 to 25 years old, 40.4% were 26 to 30 years old, 25.5% were 31 to 40 years old, and 2.1% were over 41 years old. Among the respondents, men accounted for 19.1%, and women accounted for 80.9%. The experiment was constructed as a 2 (regulatory focus: promotion vs. prevention) * 2 (emotion: cheerfulness vs. sadness) multi-factorial design. The dependent variables were willingness and behavior towards water-saving information dissemination.

#### 3.1.2. Procedure and Manipulations

The participants were told that they would be participating in a series of research studies. The specific steps were as follows. First, the participants were to asked watch a video and engage their emotions. The video consisted of a movie clip; the cheerful video came from a fragment of *Flirting Scholar*, and the sad video came from a fragment of *The Children of Huang Shi*. Second, the participants were asked to view either promotion-framed or prevention-framed water conversation information. Next, they were asked to complete a series of self-administered questionnaires, which included questions related to the dependent variables (willingness to disseminate water-saving information), manipulation checks, and demographic questions. Finally, one week later, the participants were asked to complete a questionnaire related to actual behavior water-saving information dissemination. The experiment was conducted in groups, and some gifts were given to the participants after the experiment.

#### 3.1.3. Dependent Measures

The dependent variables included willingness and actual behavior towards water-saving information dissemination. Willingness was measured by five items using a 7-point Likert scale (1 = strongly unwillingness and 7 = strongly willingness). An example question was “I would like to pass this water-saving message on to my family.” The five items were averaged to form an index of willingness to disseminate water-saving information. The participant’s actual behavior was measured by a questionnaire administered within a week of the end of the previous experiment. A sample questionnaire item was as follows: “In the past week, the number of times I have forwarded a post about water conservation to my friends is ——.”

### 3.2. Results

#### 3.2.1. Manipulation Checks

To verify the manipulation of the regulatory focus, a 7-point scale was to answer three items proposed by Poels and Dewitte (2008) [33], namely, whether the water-saving message emphasized (1) avoiding the negative or tending to the positive, (2) loss or gain, or (3) defense or improvement. The *t*-test results showed that the scores of the participants under the promotion orientation framework were significantly higher than the scores of those under the prevention orientation framework (*M_promotion_*= 5.53, *SD_promotion_*= 0.94; *M_prevention_*= 3.27, *SD_prevention_*= 1.67, *t* (79) = 5.80, *p* < 0.001). Therefore, the manipulation of the regulatory focus was successful.

To check the manipulation of emotion, a 7-point scale was used to answer six semantically differential items adopted by Hong and Lee (2010) [34], which measured positive and negative emotions. The *t*-test results showed that the participants’ scores for positive emotion were significantly higher than those for negative emotion (*M_positive_*= 5.44, *M_negative_*= 2.14, *p* < 0.001) in the positive emotion-activated group. In the negative emotion-activated group, the positive emotion scores were significantly lower than those for negative emotion (*M_positive_*= 2.02, *M_negative_*= 4.94, *p* < 0.001). These results showed that the manipulation of emotion was successful. See Table 1 for details.

#### 3.2.2. Hypothesis Testing

Next, an analysis of variance was performed. As predicted, there was a significant interaction between regulatory focus and emotion with regard to willingness to disseminate water-saving information (*F* (1,76) = 4.67, *p* < 0.05). However, no main effects were significant.

**Figure 1:**
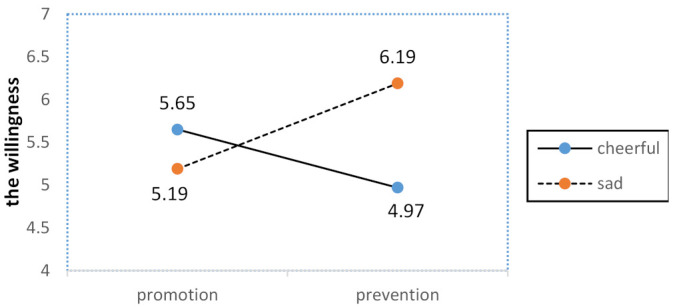
Effects of regulatory focus and emotion on willingness to disseminate water-saving information.

To further understand the interaction between emotion and regulatory focus, we performed simple effects tests. As shown in Figure 1, planned contrasts revealed that when the water-saving information was framed in a prevention focus, the participants in the sadness category reported higher levels of favorable willingness to disseminate information than did those in the cheerfulness category (*M_sad_*= 6.19 vs. *M_cheerful_* = 4.97, *F*(1,76) = 4.20, *p* < 0.05). However, when the water-saving information was framed in a promotion focus, the participants in the cheerfulness group reported no significantly different levels of willingness to disseminate information compared to those in the sadness group (*M_sad_*= 5.19 vs. *M_cheerful_*= 5.65, *F* (1,76) = 0.84, *p* > 0.05).

One week after the end of the experiment, the forwarding behavior of water-saving information was investigated.

An analysis of variance was performed. The results showed that there was a marginally significant interaction between regulatory focus and emotion in regard to the behavior of water-saving information dissemination (*F* (1,56) = 3.62, *p* = 0.06)).

As above, we also performed simple effects tests. The results revealed that when sadness was activated, the participants in prevention-focused states reported more behaviors related to information dissemination than did those in promotion-focused states (*M_prevention_*= 1.43 vs. *M_promotion_*= 1.01, *F* (1,56) = 7.45, *p* < 0.05). However, when cheerfulness was activated, the participants in promotion-focused states reported no significantly different behaviors related to information dissemination compared to those in prevention-focused states (*M_prevention_*= 1.2 vs. *M_promotion_*= 1.2, *F* (1,56) = 0, *p* > 0.05).

Our results suggest that there was a certain interplay between regulatory focus and emotion in regard to influencing willingness and behavior towards water-saving information dissemination. Specifically, when information was framed in a promotion focus, emotion had no significant influence on willingness or behavior towards water-saving information dissemination. The evidence suggests that a promotion focus caused people to pay attention to “gains” and induced “eagerness” [6], which subsequently caused them to ignore negative information (water resource shortages) and reduced their sensitivity towards relevant behaviors such as water conservation. However, when information was framed in a prevention focus, sadness fostered more willingness and behaviors towards dissemination than did cheerfulness. Accordingly, the prevention focus caused people to pay attention to “loss” and induced “vigilance” [6], which subsequently caused them to pay more attention to negative information, especially when in a state of sadness. That is, a combination of a prevention focus and sadness would appear to lead to stronger willingness or behavior towards spread water-saving information.

Although there was asymmetry in the interplay of regulatory focus and emotions, we found a trend in the findings; a prevention focus coupled with sadness was more persuasive than a prevention focus coupled with cheerfulness, while a promotion focus coupled with cheerfulness was slightly more persuasive than a promotion focus coupled with sadness. Based on these results, we speculated that there might be a fit between the regulatory focus and emotion. The fit conditions consisted of a promotion focus with cheerfulness or a prevention focus with sadness. The nonfit conditions consisted of a promotion focus with sadness or a prevention focus with cheerfulness. In the next study we test the second set of hypotheses.

## 4. Study 2

Study 2 was developed to test the regulatory fit. We predicted that individuals who experienced regulatory fit (promotion focus/cheerfulness or prevention focus/sadness) would show higher levels of favorable willingness or behaviors towards disseminating water-saving information than would those who experienced regulatory nonfit (promotion focus/sadness or prevention focus/cheerfulness) (Hypothesis 2).

### 4.1. Participants and Design

A total of 36 undergraduates from Zhejiang University took part in the experiment. None of the participants had previously participated in a similar experiment. The experiment was designed as a single-factor experiment. The participants were randomly divided into two groups. In each group, the participants were given two experimental interventions with different combinations of conditions at intervals of one week. One group received experimental conditions such as promotion focus/cheerfulness and prevention focus/sadness (fit conditions), while the other group received experimental conditions such as promotion focus/sadness and prevention focus/cheerfulness (nonfit conditions). The manipulations of the independent and dependent measures were similar to those used in study 1, with the exception that study 2 included a measure of the participants’ attitudes towards water conservation.

### 4.2. Results

**Figure 2:**
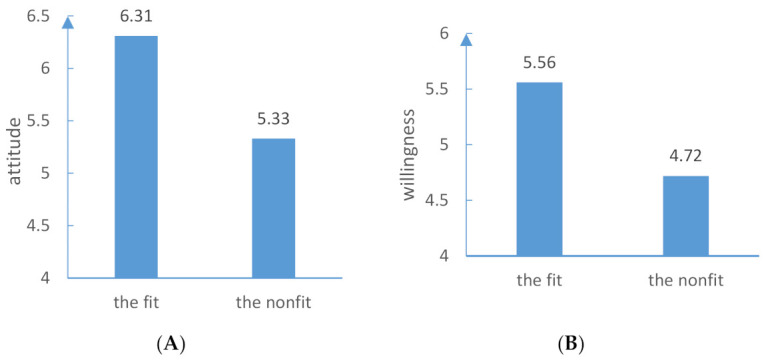
(**A**) The matching effects of regulatory focus and emotion on attitude towards water-saving information. (**B**) The matching effects of regulatory focus and emotion on willingness to spread water-saving information.

Attitude. An independent sample *t*-test showed that the scores for attitude towards water-saving information for the fit group were significantly higher than those for the nonfit group, indicating that the matching of emotion and regulation orientation has a significant impact on an individual’s attitude towards water-saving information (*M_fit_*= 6.31, *SD_fit_*= 0.79; *M_nonfit_*= 5.33, *SD_nonfit_*= 1.24; *t* = 2.77, *p* < 0.05). The evidence is shown in Figure 2A.

Willingness. A *t*-test showed that compared to the nonfit condition, the fit group had slightly higher scores on the intention to spread water-saving information, and the difference between the two groups reached a marginal level of significance (*Mfit* = 5.56, *SD_fit_*= 1.15; *M_nonfit_*= 4.72, *SD_nonfit_*= 1.36; *t* = 1.93, *p* = 0.06). See Figure 2B.

Behavior. Independent sample *t*-tests showed that there was no significant difference in the water-saving information dissemination behaviors between the fit group and the nonfit group; however, the number of water-saving information dissemination behaviors in the fit group tended to be slightly greater than that in the nonfit group (*M_fit_*= 2.14, *SD_fit_*= 1.15; *M_nonfit_*= 1.63, *SD_nonfit_*= 1.17; *t* = 1.15, *p* > 0.05).

## 5. Discussion

The objective of this research was to investigate how the effects of regulatory focus and emotion are activated in regard to fostering willingness and behavior towards water-saving information dissemination. By using the information framework method to manipulate regulatory focus, two sub-studies were carried out. Study 1 showed that there was an interactive effect of regulatory focus and emotion present in regard to the willingness to disseminate water-saving information. Specifically, under the condition of a prevention focus, people were more conducive to the dissemination of water-saving information when they were sad than when they were cheerful, while under the condition of a promotion focus, the influence of emotion on willingness to disseminate water-saving information was not significant. However, promotion-focused individuals had a tendency to have a higher level of willingness to disseminate water-saving information when they were cheerful compared to when they were sad. On the basis of study 1, study 2 further explored the matching effect of emotion and regulatory focus. A longitudinal study was used to intervene in two groups of participants (fit vs. nonfit). The findings indicated that the individuals in the fit group had more favorable attitudes towards water conservation and behavioral intentions to spread water-saving information compared to those in the nonfit group. Prior work has suggested that promotion-focused individuals more easily experience cheerful/disappointed emotions, while prevention-focused individuals more easily experience calm/sad emotions [9]. The regulatory focus and corresponding specific emotions could form a matching effect, which might affect the subsequent psychology and behavior and thus produce a more positive attitude [24].

Furthermore, the current research extends the literature on regulatory focus and emotion. Prior studies have found that individuals in a promotion-focused condition are more affected by emotion than are those in a prevention-focused condition [35]. A happy mood has a greater influence on individuals with a promoting orientation than on those who are in a sad mood [36]; however, in the current research, we used information related to water saving as the experimental materials, and we found that the sadness emotion had a greater impact on individuals with a prevention focus than on those with a promotion focus. Why was there such a difference? This result may have been caused by different associations being stimulated by experimental materials. Previous studies have examined how people’s pre-existing cognitive structures affect their interpretation and integration of new information [37]. We speculate that the experimental materials (positive materials) might have made the participants yearn for such an orientation, such that the experimental materials can start to promote orientation internally. When the promotion orientation is underway, the individual promotion orientation should be more prominent; that is, the promotion orientation is strengthened in the promotion-focused group, while the prevention orientation is attenuated in the prevention-focused group. In contrast, in the current research, when the participants were faced with water-saving information materials (negative materials), they generally did not have good expectations about water resources or their utilization status, which led to avoidance tendencies. That is, the water-saving materials inherently activated preventive orientation. When a prevention focus is primed, an individual’s prevention orientation should be highlighted. Accordingly, the prevention orientation was strengthened while the promotion orientation was attenuated in the promotion-focused group. When the material information is consistent with the temporary start of the situation, the influence effect may be more prominent. Consistent with Aaker and Lee (2001) [38], when the representation framework of information is consistent with the contextually initiated self-concept and the long-formed self-concept respected by culture, the influence effect is best. This is also consistent with the earlier study of Bargh, Bond, Lombardi, and Tota (1986) [39], which found that the accessibility of personality, coupled with the influence of matching situations, will enhance the experimental treatment effect.

The current results show that emotions play important roles in the influence of regulatory focus on people’s attitudes and behaviors towards water-saving information. However, previous studies have drawn different conclusions about the effects of emotions. For example, it has been suggested that emotions trigger heuristic processing, thereby leading to assimilation effects on people’s judgements. That is, while they are experiencing a positive emotion, people are likely to rate a positive outcome more positively, and while they are experiencing a negative emotion, people are likely rate a negative outcome more negatively [40,41]. Some researchers have found that this is not always the case. For example, some scholars have found that negative emotions do not always lead to negative reactions. Tan and Forgas (2010) found that happiness increased selfishness, while sadness produced greater fairness [42]. Negative emotions have always been found to produce more favorable results than positive emotions [43]. These results are consistent with our research conclusion. Under the condition of prevention orientation, sad emotions are more conducive to people’s positive attitude towards water conservation and their willingness to disseminate water-saving information.

It may be possible to reconcile the differences between these studies according to the emotion consistency effect. That is, when the valence of emotion matches the valence of the target stimulus, a favorable evaluation will be produced. Specifically, positive emotions lead to more favorable assessments of positive information, while negative emotions lead to more favorable assessments of negative information [21]. Therefore, for positive target stimulations, positive emotions may improve the attractiveness of target stimulation; however, for some difficult social problems, such as water resource shortages and other related problems, negative emotions may be beneficial because these problems can trigger emotional resonance and thus produce more altruistic behaviors [44].

## 6. Conclusions

Water saving is regarded as one of the effective ways to protect water resources. This study uses the experimental method to discuss how to promote the protection of water resources. The results are as follows: Under the condition of sad emotions, individuals with a prevention focus had a higher level of willingness to disseminate water-saving information than did those with a promotion focus and thus produced more positive behaviors towards water-saving information dissemination. Compared to those in the nonfit group regarding emotions and regulatory focus, the individuals in the fit group formed more favorable water-saving attitudes and showed slightly higher levels of willingness to disseminate water-saving information.

The findings of this study reveal some strategic ways in which appropriate advertising message strategies can be used to persuade people to save water. Based on these findings, advertising messages should consider the regulatory focus of the audience. If communicators pay more attention to the matching role of emotion and regulatory focus when advocating water-saving behavior, the message of water-saving activities can be created and deployed more effectively. Therefore, choosing an appropriate stimulus to trigger happiness or sadness is very important for the dissemination of water saving information. For example, water-saving advertising messages using a prevention-focused framework may be more effective if they are transmitted in programs most likely to trigger sad emotional states. On the other hand, when the target audience is in a cheerful emotional state, they should emphasize a promotion-focused framework that attempts to exert influence on the attitude and willingness to save water.

Our research can provide some points of reference for research fields’ related environmental protection and other fields of application. However, there are still some deficiencies in the current study. First, we adopted a primed regulatory focus instead of a chronic regulatory focus; thus, it is not clear whether chronic regulatory orientation has the same effects. In addition, it must be questioned whether the temporary primed orientation is influenced by the chronic regulatory orientation? Therefore, a future follow-up study might consider the effects of chronic regulatory orientation and discuss the interactive effects between primed and chronic regulatory focus. Second, our study used the self-report method to examine the experimental treatment effect. Future research should consider other methods, such as the realistic direct assessment of actual behaviors.

## Figures and Tables

**Table 1:** *t*-test of the manipulation of emotions.

Manipulation	Emotions	*M*	*SD*	*t*	df	*p*
Positive	Positive	5.44	1.17	12.31	77	0.000
	Negative	2.14	1.21			
Negative	Positive	2.02	1.31	−11.08	78	0.000
	Negative	4.94	1.05			

## Data Availability

The data presented in this study are available on request from the corresponding author.

## References

[1] Hutton G., Chase C. (2016). The Knowledge Base for Achieving the Sustainable Development Goal Targets on Water Supply, Sanitation and Hygiene. Int. J. Environ. Res. Public Health.

[2] Low K.G., Grant S.B., Hamilton A.J., Gan K., Saphores J., Arora M., Feldman D.L. (2015). Fighting drought with innovation: Melbourne’s response to the millennium drought in southeast australia: Fighting drought with innovation. Wires. Water.

[3] Robins S. (2019). ‘Day zero’, hydraulic citizenship and the defence of the commons in cape town: A case study of the politics of water and its infrastructures (2017−2018). J. S. Afr. Stud..

[4] Koop S., Van Dorssen A., Brouwer R. (2019). Enhancing domestic water conservation behaviour: A review of empirical studies on influencing tactics. J. Environ. Manag..

[5] Olivier D.W., Xu Y. (2019). Making effective use of groundwater to avoid another water supply crisis in Cape Town, South Africa. Hydrogeol. J..

[6] Cesario J., Corker K.S., Jelinek S. (2013). A self-regulatory framework for message framing. J. Exp. Soc. Psychol..

[7] Cho H., Boster F.J. (2008). Effects of Gain Versus Loss Frame Antidrug Ads on Adolescents. J. Commun..

[8] Cesario J., Grant H., Higgins E.T. (2004). Regulatory Fit and Persuasion: Transfer From “Feeling Right”. J. Personal. Soc. Psychol..

[9] Higgins E.T. (1997). Beyond pleasure and pain. Am. Psychol..

[10] Higgins E.T. (2002). How self-regulation creates distinct values: The case of promotion and prevention decision-making. J. Consum. Psychol..

[11] Idson L.C., Liberman N., Higgins E.T. (2004). Imagining How You’d Feel: The Role of Motivational Experiences from Regulatory Fit. Personal. Soc. Psychol. Bull..

[12] Chung J. (2020). Effect of Quality Uncertainty, Regulatory Focus, and Promotional Strategies on Perceived Savings for Sustainable Marketing. Sustainability.

[13] Higgins E.T. (2000). Making a good decision: Value from fit. Am. Psychol..

[14] Huang M., Wang Y., Liao J., Liu M. (2017). Mixed effects of inconsistent reviews on consumers: The moder-ating roles of product attributes and regulatory focus. Acta Psychol. Sinica.

[15] Friedman R.S., Förster J. (2001). The effects of promotion and prevention cues on creativity. J. Personal. Soc. Psychol..

[16] Pham M.T., Avnet T. (2004). Ideals and Oughts and the Reliance on Affect versus Substance in Persuasion. J. Consum. Res..

[17] Pham M.T., Avnet T. (2009). Contingent reliance on the affect heuristic as a function of regulatory focus. Organ. Behav. Hum. Decis. Process..

[18] Zhu R., Meyers-Levy J. (2007). Exploring the Cognitive Mechanism that Underlies Regulatory Focus Effects. J. Consum. Res..

[19] Wagner D.G., Higgins E.T., Sorrentino R.M., Sorrentino R.M., Higgins E.T. (1986). Cognition, emotion, and action. Handbook of Motivation and Cognition: Foundations of Social Behavior.

[20] Labroo A.A., Patrick V.M. (2009). Psychological Distancing: Why Happiness Helps You See the Big Picture. J. Consum. Res..

[21] Wegener D.T., Petty R.E., Klein D.J. (1994). Effects of mood on high elaboration attitude change: The mediating role of likelihood judgments. Eur. J. Soc. Psychol..

[22] Lench H.C., Flores S.A., Bench S.W. (2011). Discrete emotions predict changes in cognition, judgment, experience, behavior, and physiology: A meta-analysis of experimental emotion elicitations. Psychol. Bull..

[23] Shah J., Higgins E.T. (2001). Regulatory concerns and appraisal efficiency: The general impact of promotion and prevention. J. Personal. Soc. Psychol..

[24] Cornelis E., Cauberghe V., De Pelesmacker P. (2014). Regulatory congruence effects in two-sided advertising. Eur. J. Mark..

[25] Koenig A.M., Cesario J., Molden D.C., Kosloff S., Higgins E.T. (2009). Incidental Experiences of Regulatory Fit and the Processing of Persuasive Appeals. Personal. Soc. Psychol. Bull..

[26] Idson L.C., Liberman N., Higgins E. (2000). Distinguishing Gains from Nonlosses and Losses from Nongains: A Regulatory Focus Perspective on Hedonic Intensity. J. Exp. Soc. Psychol..

[27] Lee A.Y., Aaker J.L. (2004). Bringing the frame into focus: The influence of regulatory fit on processing fluency and persuasion. J. Personal. Soc. Psychol..

[28] Malaviya P., Sternthal B. (2009). Parity Product Features Can Enhance or Dilute Brand Evaluation: The Influence of Goal Orientation and Presentation Format. J. Consum. Res..

[29] Wang J., Lee A.Y. (2006). The Role of Regulatory Focus in Preference Construction. J. Mark. Res..

[30] Spiegel S., Grant-Pillow H., Higgins E.T. (2004). How regulatory fit enhances motivational strength during goal pursuit. Eur. J. Soc. Psychol..

[31] Brockner J., Higgins E. (2001). Regulatory Focus Theory: Implications for the Study of Emotions at Work. Organ. Behav. Hum. Decis. Process..

[32] Holler M., Hoelzl E., Kirchler E., Leder S., Mannetti L. (2008). Framing of information on the use of public finances, regulatory fit of recipients and tax compliance. J. Econ. Psychol..

[33] Poels K., Dewitte S. (2008). Hope and self-regulatory goals applied to an advertising context: Promoting prevention stimulates goal-directed behavior. J. Bus. Res..

[34] Hong J., Lee A.Y. (2010). Feeling Mixed but Not Torn: The Moderating Role of Construal Level in Mixed Emotions Appeals. J. Consum. Res..

[35] Wang X., Zheng Q., Wang J., Gu Y., Li J. (2020). Effects of regulatory focus and emotions on information preferences: The affect-as-information perspective. Front. Psychol..

[36] Baek T.H., Reid L.N. (2013). The Interplay of Mood and Regulatory Focus in Influencing Altruistic Behavior. Psychol. Mark..

[37] Petty R.E., Cacioppo J.T. (1986). The elaboration likelihood model of persuasion. Adv. Exp. Soc. Psychol..

[38] Aaker J.L., Lee A. (2001). “I” seek pleasures and “We” avoid pains: The role of Self-Regulatory goals in information processing and persuasion. J. Consum Res..

[39] Bargh J.A., Bond R.N., Lombardi W.J., Tota M.E. (1986). The additive nature of chronic and temporary sources of construct accessibility. J. Personal. Soc. Psychol..

[40] Petty R.E., Schumann D.W., Richman S.A., Strathman A.J. (1993). Positive mood and persuasion: Different roles for affect under high and low elaboration conditions. J. Personal. Soc. Psychol..

[41] Teeny J.D., Siev J.J., Brinol P., Petty R.E. A review and conceptual framework for understanding personalized matching effects in persuasion. J. Consum. Psychol..

[42] Tan H.B., Forgas J.P. (2010). When happiness makes us selfish, but sadness makes us fair: Affective influences on interpersonal strategies in the dictator game. J. Exp. Soc. Psychol..

[43] Carlson M., Miller N. (1987). Explanation of the relation between negative mood and helping. Psychol. Bull..

[44] Putrevu S. (2014). Effects of Mood and Elaboration on Processing and Evaluation of Goal-Framed Appeals. Psychol. Mark..

